# Comparison of MT-PCR with Quantitative PCR for Human Bocavirus in Respiratory Samples with Multiple Respiratory Viruses Detection

**DOI:** 10.3390/diagnostics13050846

**Published:** 2023-02-23

**Authors:** Maja Mijač, Sunčanica Ljubin-Sternak, Irena Ivković-Jureković, Jasmina Vraneš

**Affiliations:** 1 Molecular Microbiology Department, Dr. Andrija Štampar Teaching Institute of Public Health, 10000 Zagreb, Croatia; 2 Medical Microbiology Department, University of Zagreb School of Medicine, 10000 Zagreb, Croatia; 3 Department of Pulmonology, Allergy, Immunology and Rheumatology, Children’s Hospital Zagreb, 10000 Zagreb, Croatia; 4 Faculty for Dental Medicine and Healthcare, School of Medicine, Josip Juraj Strossmayer University of Osijek, 31000 Osijek, Croatia

**Keywords:** human bocavirus, children, respiratory tract, viral load, MT-PCR

## Abstract

Human bocavirus (HBoV) is an important respiratory pathogen, especially in children, but it is often found in co-detection with other respiratory viruses, which makes the diagnostic approach challenging. We compared multiplex PCR and quantitative PCR for HBoV with multiplex tandem PCR (MT-PCR) in 55 cases of co-detection of HBoV and other respiratory viruses. In addition, we investigated whether there is a connection between the severity of the disease, measured by the localization of the infection, and amount of virus detected in the respiratory secretions. No statistically significant difference was found, but children with large amount of HBoV and other respiratory virus had a longer stay in hospital.

## 1. Introduction

Human bocavirus, a small non-enveloped DNA virus that belongs to the Parvoviridae family, was first discovered 2005 by Thomas Allander in the nasopharyngeal samples of children with acute respiratory illness by random molecular screening [1]. Later, three more viruses were found in fecal samples and named human bocavirus 2, 3, and 4, to differentiate them from the first isolated subtype, named human bocavirus 1 (HBoV1); their connection with gastrointestinal infections was investigated [2]. Human bocavirus 1 (HBoV1, further in the text defined as HBoV) is recognized as an important respiratory pathogen, especially in children, which is connected to rhinitis, acute otitis media, pneumonia, bronchiolitis, and asthma exacerbations [3,4,5,6,7]. HBoV is often found in combination with other respiratory pathogens, with rate of co-detection between 8.3% to 100% [2]. It can also be found in asymptomatic children, probably because of prolonged shedding [8] and possible persistence [9]. HBoV1 has only been found to be capable of infection in one in vitro system, which is the human airway epithelium (HAE) cultured at an air–liquid interface (HAE-ALI) made from differentiated (nondividing) epithelial cells. The other system mentioned is the HEK 293 cell line, although replication is possible after the transfection of a pIHBoV1 plasmid that holds the complete double-stranded genome. Other cell lines (HEp-2, Vero MCR-5, etc.) are not susceptible to the virus, probably due to the lack of a specific receptor, which has yet to be discovered [2,10,11]. Due to difficulties of growth in cell culture and the fact that serology is complicated with the existence of the other three types of virus, the diagnostic approach for the detection of HBoV is almost exclusively by molecular methods [2,3,12], mainly by multiplex PCR, as it is part of the standard respiratory panels. One of proposed methods is quantitative PCR, which provides more information about viral load of HBoV [3,12]. For quantitative PCR, a viral load higher than 10^4^ copies/mL is thought to be important [13,14,15], although some studies suggest a higher viral load as a cut off for acute viral infection, more than 10^6^ copies/mL. The advancement and wider use of new molecular techniques during the pandemic era offer new opportunities for investigating the virus. One of those methods is multiplex tandem PCR (MT-PCR) [16]. MT-PCR is a nested PCR, which employs two sequential PCR steps. Step 1 is a short multiplex pre-amplification reaction using primers homologous to all targets offered by the product. Each pair of nested primers are specific to one target. The next step contains primer pairs for every target offered by the kit and Step 2 primers are designed to be “nested inside” the Step 1 primers, which increases the sensitivity and specificity of the assay. The next step is real-time PCR for each target. MT-PCR is not a traditional quantitative method as it cannot determine the viral load in terms of copies per mL, but it can provide information on the amount of viruses by comparing them to a standard amount (spike control). It presents results in the form of intensity, a 5-star rating system for each targeted pathogen. The aim of this work is to compare MT-PCR with multiplex PCR and quantitative PCR for the detection of HBoV, with an emphasis on co-detection of HBoV with other respiratory viruses.

## 2. Materials and Methods

### 2.1. Inclusion Criteria and Sample Collection

In the period from May 2017 to March 2012, we collected samples from patients aged 0 to 18 years old who were hospitalized in two Croatian hospitals, Children’s Hospital Zagreb and General Hospital Karlovac. The inclusion criteria were acute respiratory disease, proposed viral etiology, lasting no longer than five days, and requiring hospitalization.

Samples collected for viral detection were nasopharyngeal and pharyngeal flocked swabs from each patient, combined, and placed in viral transport medium (UTM^®^, Copan, Brescia, Italy), and stored in laboratory at −80 °C until tested.

**Figure 1:**
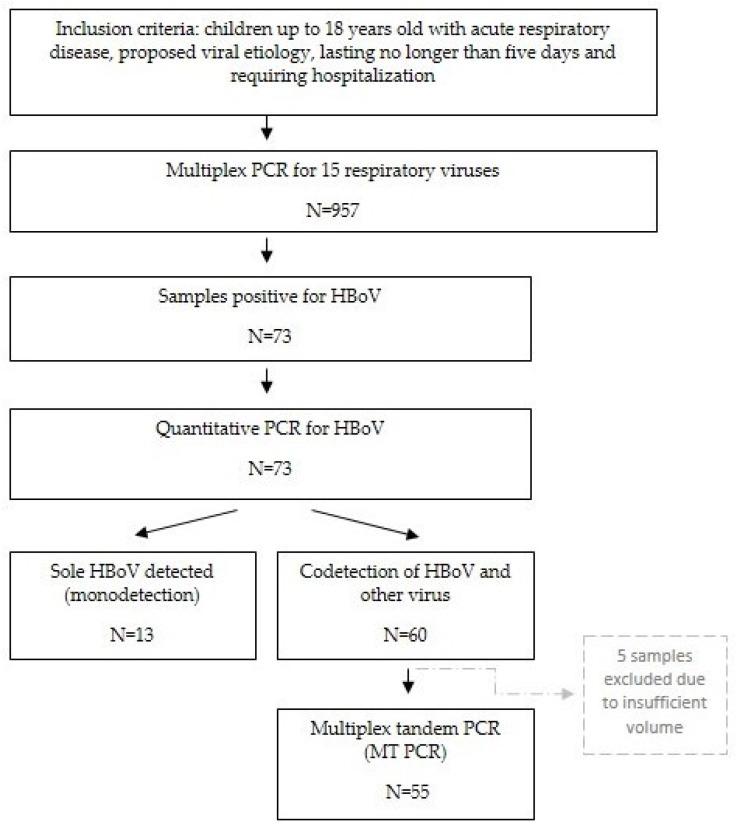
Conceptual framework of the research.

The conceptual framework of the research is presented in Figure 1.

### 2.2. Multiplex PCR for 15 Respiratory Viruses

After isolation of viral DNA and RNA from viral transport medium, which was performed using the Ribospin^TM^ vRD Kit (GeneAll Biotechnology, Seoul, Korea), multiplex RT-PCR for 15 respiratory viruses, including adenovirus (AdV), human coronavirus (HCoV) 229E/Nl63, HCoV OC43, parainfluenza virus (PIV) types 1-4, RV groups A/B/C, RSV type A and B, influenza (Flu) type A and B, human bocavirus (HBoV), human metapneumovirus (HMPV), and human enterovirus (HEV), was performed using the Seeplex^®^ RV15 OneStep ACE Detection (Seegene Inc., Seoul, Republic of Korea). Amplification was performed with a thermal cycler GeneAmp^®^ 9700 PCR System (Applied Biosystems, Foster City, United States). Detection of PCR products was done by microchip electrophoresis on the MCE^®^-202 MultiNA device (Shimadzu, Kyoto, Japan). Seeplex^®^ RV15 OneStep ACE Detection includes two internal controls: one is PCR control, in order to check the presence of substances that may interfere with PCR amplification. The other control is called the whole process control (WPC) and includes the human RNase P target as an internal control to check the entire experimental process from nucleic acid extraction to RT-PCR.

### 2.3. Quantitative PCR for HBoV

Quantitative PCR for samples positive for human bocavirus was performed using the LightMix^®^ Modular Bocavirus kit with a LightCycler 480^®^ System (Roche Diagnostics, Rotkreuz, Switzerland). PCR primers BoF (GGAAGAGACACTGGCAGACAA), BoR (GGGTGTTCCTGATGATATGAGC), and a hydrolysis probe BoP (Cy5-CTGCGGCTCCTGCTCCTGTGAT-BHQ2) targeting the NP-1 gene of HBoV have been described previously [6,17].

### 2.4. Multiplex Tandem PCR

Samples in which HBoV was detected in combination with at least one other respiratory virus were further analyzed on High-Plex 24 System (AusDiagnostics, Mascot, NSW, Australia), based on the principle of Multiplex Tandem PCR method (MT-PCR) using the respiratory viruses 16 well test, which targets 16 viruses: influenza A, influenza B, RSV, rhinovirus/enterovirus, enterovirus, human bocavirus, human parainfluenza viruses 1-4, human parechovirus, human adenoviruses, human coronaviruses, and human metapneumovirus. The test consists of several controls: positive and negative control, sample adequacy and human DNA control, suitable nucleic acid control, and sample inhibition and instrument function control to ensure the whole process works well.

### 2.5. Clinical Data Collection

Demographic and clinical data were collected by a retrospective review of patient medical records: including duration of hospitalization, antimicrobial use record, need for oxygen supplementation, need for mechanical ventilation, routine microbiology studies, and data from chest radiographs, which were taken only if necessary, in order to avoid unnecessary X-rays.

## 3. Results

### 3.1. Multiplex PCR Results

In the period from May 2017 to March 2012, we collected 957 samples. Using multiplex PCR, human bocavirus was found in 73 samples (73/957; 7.6%), in 41 male and 32 female patients. The median age of the patients positive for HBoV was 1.36 years (the youngest child was 7 days old and the oldest was 15 years old). In 60 cases (60/73, 82%), HBoV was detected in combination with at least one other respiratory virus, and in 13 cases, sole HBoV was discovered.

### 3.2. Quantitative PCR Results

Quantitative PCR for HBoV samples was performed for 73 samples positive for HBoV and it was successful in 65/73 samples. Eight (8/73) samples were negative in this assay, in all of them HBoV was detected with other viruses on multiplex PCR. Viral load ranged from 9.71 to 2.14 × 10^7^ copies/mL (median 3.91 × 10^4^ copies/mL). In 39 (39/73) samples, a high concentration (more than 10^4^ copies/mL) was found.

### 3.3. Comparison between Multiplex PCR and MT-PCR

**Figure 2:**
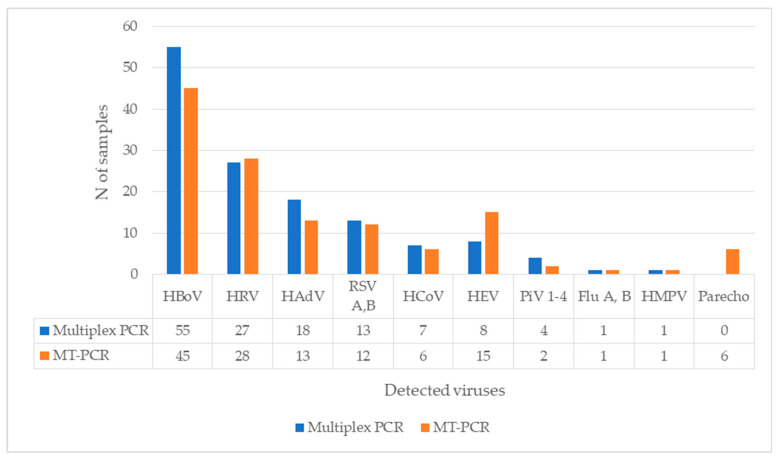
Codetection of HBoV with other respiratory viruses, a comparison of multiplex PCR and MT-PCR (*N* of samples = 55).

From 60 samples with co-detection, 55 were suitable for further analysis with the MT-PCR method. Of the 55 positive samples for HBoV and other respiratory virus on multiplex PCR, HBoV was present in 45 (45/55) samples on MT-PCR. The most common virus found in co-detection with both methods was human rhinovirus, followed by human adenovirus and RSV. A comparison of results for each virus are presented in Figure 2. Note that multiplex PCR does not consist of primers for parechovirus, while MT-PCR reported enterovirus (EV) in 15 samples, all of them were also positive on the rhinovirus assay (RV/EV).

HRV—human rhinovirus, AdV—human adenovirus, RSV A B—Respiratory syncytial virus type A and B, HCoV—seasonal human coronavirus, HEV—human enterovirus, PIV 1-4—parainfluenza virus 1-4, HMPV—human metapneumovirus, Flu A B—influenza type A and B, parecho-parechovirus.

In four samples, sole HBoV was detected. Results of the multiplex PCR and quantitative HBoV PCR for those samples are shown in Table 1.

In 10 samples, HBoV was not detected (one sample was negative for all tested viruses). All of them had very low concentration of HBoV or were negative on quantitative PCR, as it is shown in Table 2.

### 3.4. Comparison of Quantitative PCR for HBoV and MT PCR

**Figure 3:**
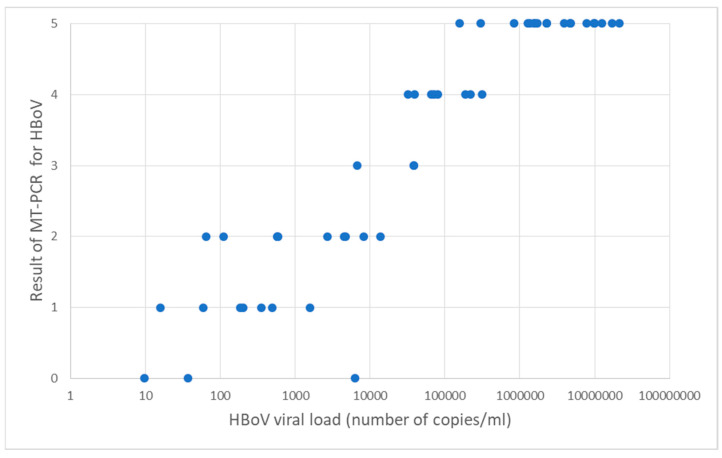
Comparison of 5-star based representation on MT-PCR (intensity) and quantitative HBoV PCR: *x* axis represents concentration of HBoV by quantitative PCR and *y* axis number of stars on MT-PCR test.

A high HBoV concentration (more than 10^4^ copies/mL) was found in 28/55 samples. Although MT-PCR does not express absolute quantitative concentrations of viruses in a sample, it can provide additional information how much of the targeted gene is present in the sample by 5-star-based representation marked as intensity. We compared results of HBoV quantitative PCR with results on MT-PCR. Of 28 samples with a high concentration on quantitative PCR (more than 10^4^ copies/mL), 25 samples were marked with 4–5 stars on MT-PCR. A comparison of the results are presented in Figure 3.

### 3.5. Characterization of Co-Detections and Comparison of the Amount of HBoV and Other Viruses

**Figure 4:**
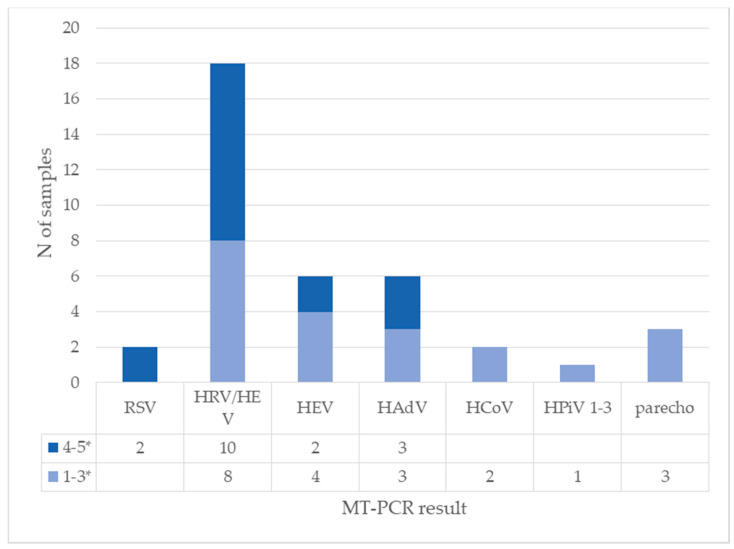
Detection of other viruses on MT-PCR in samples with high concentration of HBoV on quantitative PCR (*N* = 28). Results of MT-PCR are expressed by 5-star representation marked as intensity (1–3* and 4–5*) In some samples, more than one other virus is present.

**Figure 5:**
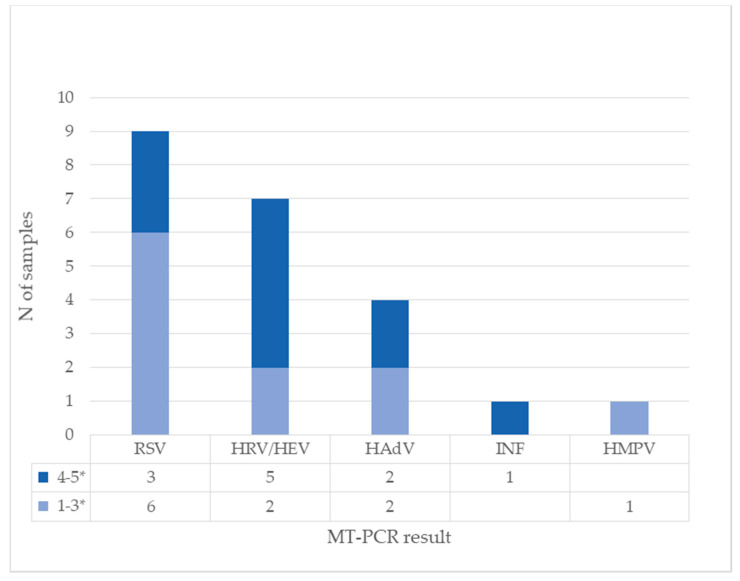
Detection of other viruses on MT-PCR in samples with low concentration of HBoV on quantitative PCR (*N* = 27). Results of MT-PCR are expressed by a 5-star-based representation marked as intensity (1–3* and 4–5*). In some samples, more than one other virus is present.

Next, the quantitative nature of MT-PCR potentially allows us to compare relative quantity of viruses in cases of co-infection. We analyzed the amount of other viruses in samples with HBoV to see if there is a difference between samples with high and low HBoV concentrations. In 28 samples with a high concentration of HBoV on quantitative PCR (more than 10^4^ copies/mL), four viruses were present in a high amount (marked with 4–5 stars on MT-PCR): RSV, human rhinovirus, human enterovirus, and human adenovirus. Other viruses were detected in small concentrations (marked 1–3 stars on MT-PCR). The results are presented in Figure 4 and Figure 5.

### 3.6. Clinical Relevance of Different Amounts of HBoV in Cases of Co-Detection

Clinically, of 55 analyzed patients with co-infection, 23 had infections in the upper respiratory tract (URTI) and 32 had a lower respiratory tract infection (LTRI). The average duration of hospitalization was 6.5 days. Eight patients needed oxygen supplementation and 33 of them received antibiotics. Additional microbiological investigation (nasopharyngeal and throat swab, urine sample, blood culture and serology for *M. pneumoniae* and *C. pneumoniae*) for each child was undertaken upon clinical indication. In one child, *S. pneumoniae* was grown in blood culture, one child had *H. influenzae* in the nasopharyngeal swab, and one child had *S. pyogenes* group A in a throat swab. The other children had no bacteriological isolates and received antimicrobial therapy empirically. Radiologic imaging of the lung was indicated in 36/55 patients and in 23 patients’ pathologic findings were found. The average body temperature was 37.2 °C. The median CRP was 28.15 mg/L and median leukocyte count was 7.08 × 10^9^/L.

Furthermore, we investigated if there was a difference between the localization of infection in the respiratory tract (URTI and LRTI) in relation to relative amount of viruses in sample. For that purpose, we divided patients in four groups:

A- Samples with a high concentration of HBoV (more than 10^4^ copies/mL in quantitative PCR) and a high score of other viruses (intensity 4–5 stars on MT-PCR);
B- Samples with a high concentration of HBoV (more than 10^4^ copies/mL in quantitative PCR of and a low score of other viruses (intensity 1–3 stars on MT-PCR);
C- Low concentration of HBoV (less than 10^4^ copies/mL in quantitative PCR) or negative and high score of other viruses (intensity 4–5 stars on MT-PCR);
D- Low concentration of HBoV (less than 10^4^ copies/mL in quantitative PCR) or negative and low score of other viruses (intensity 1–3 stars on MT-PCR).

The results are presented in Table 3. There was no statistically significant difference between groups on the chi-square test (*p* = 0.53).

## 4. Discussion

The last three years of the pandemic has pointed viral respiratory infections as a focus of medical and social interest, thus, understanding their etiology is of outmost importance. Since its first discovery 2005, HBoV is recognized as an important respiratory pathogen in the child population. However, diagnostic limitations and lack of animal models are still leaving questions about clinical significance and pathogenesis of this infection open. Our study revealed a high prevalence of HBoV (7.6%) among children hospitalized for acute respiratory infection during a four-year period, which points to it as the fifth most frequently detected virus in respiratory tract samples after RV, RSV, AdV, and PiV [18]. The results are in accordance with other prevalence studies. Guido et al. [2] estimated a global HBoV prevalence of 6.3 and 5.9% in respiratory and gastrointestinal infections, respectively, and a large systematic analysis from 2022 found that the HBoV prevalence in Europe varied from 2.0 to 45.69% with a pooled estimate of 9.57% [19]. Additionally, a high occurrence of co-detection with other respiratory viruses was present in our study (82.2%), which is also a well-documented characteristic of HBoV. In a review that included 311 studies from 50 countries all over the world performed between 2005 and 2016, HBoV-positivity ranged from 8.3% to 100%, with total co-infection estimates in the 193 studies covered of 52.4% [2]. It is probably result of prolonged shedding from the nasopharynx, found both in immunocompetent and immunocompromised children [2,20,21], and possibly persistence [2,22]. The diagnosis of this virus and the interpretation of its test results, often in conjunction with other respiratory viruses, is controversial. It is clear that relying solely on qualitative PCR is not sufficient for a definitive diagnosis due to a high rate of co-detection with other respiratory viruses. Other proposed methods are quantitative PCR, mRNA detection [3,23,24], and serology, which are complicated with the existence of the three other types of viruses, HBoV 2-4, causing immunological cross-reactivity and a phenomena called original antigenic sin [25]. Quantitative PCR suggests that a viral load above 10^4^ copies/mL is significant; however, some research indicates that for acute viral infections, a cut off of over 10^6^ copies/mL is more appropriate [3,16,26]. A study that compared performances between mRNA detection and quantitative PCR showed that quantitative PCR has sensitivity of 100%, specificity of 93–99%, and positive predictive values (PPV) of 56–87%. When serology was used, the standard sensitivity of quantitative PCR was 81%, specificity was 92%, and the PPV was 87% [24].

In the era of pandemics, we are witnessing to broader application of molecular methods and the wider use of automated and semi-automated systems, which opens doors for easier diagnostic approach to this virus.

In this work, we compared classic quantitative PCR for HBoV with MT-PCR, which is one of those methods.

Although MT-PCR does not provide an exact number of copies (viral load), as does classic quantitative PCR, it is naturally a quantitative method [27] and expresses results as intensity with a 5-star-based representation for every targeted pathogen. We compared those results for human bocavirus with results of quantitative PCR and high congruence was present, 25/28 samples with a high concentration of HBoV on quantitative PCR (more than 10^4^ copies/mL) had 4–5 stars on the MT-PCR intensity report for this virus. Ten samples positive for HBoV on multiplex PCR were negative on the MT-PCR test, but for seven of them, quantitative PCR was also negative, and two samples had very low number of copies of HBoV, at less than 50 copies. MT-PCR has endogenous control of extraction and amplification, named Sample Adequacy Control, which targets a human reference gene as an indicator of suitability of nucleic acid extract and it was appropriate for all 10 negative samples. Sample inhibition control, named SPIKE, was also positive, so the negative MT-PCR result could not be explained with extraction or amplification failure. The low level of viral DNA in the sample may have resulted from degradation over time due to extended freezing. Next, the majority of practitioners still approach respiratory infections assuming single pathogen etiology. However, sensitive molecular methods have discovered that multiple pathogens can be detected, even in cases of influenza or respiratory syncytial virus infection [28], but the influence of such co-detection on severity of respiratory diseases is still unclear [29,30]. In order to highlight cases of co-detection in which HBoV is present in combination with other viruses, we divided the patients in four groups to see is there a difference in clinical presentation between them. It would be expected that patients with high amount of human bocavirus and high intensity of other respiratory virus had a more severe clinical presentation, as some studies suggest [14,31,32], and more often develop infection of the lower respiratory system (LRTI). Moreover, Moesker et al. [33] reported a higher viral load in HBoV-positive patients admitted to an intensive care unit. Contrarily, other studies are questioning such findings, including one multicentric study which compared viral load in the upper respiratory tract of children with pneumonia and age-matched community controls and the results were equivocal [34]. In our study, we did not find a significant difference in the localization of infection in the respiratory tract between the groups of patients divided according to the amount of detected viruses in nasopharyngeal/pharyngeal samples. However, we did find that children from group A (high amount of both HBoV and other virus) had a slightly longer average stay in hospital, 6.64 days, respectively, compared to the other three groups, where the average hospitalization days were 5.38, 4.25, and 5.75 days for group B, C, and D, repsectively. The average stay in hospital for children with mono-infection with HBoV was 5.75 days.

The study has a few limitations, including the relatively small sample size that hinders a detailed analysis of the clinical differences between groups. Further research, ideally with multiple centers involved, is needed to better understand the clinical outcomes of HBoV co-infections with other viruses.

Subsequently, a complete analysis of all samples with all three methods was not possible. Specifically, five samples detected with co-detection could not undergo further analysis using MT-PCR due to insufficient sample volume. As a result, the final analysis was based on 55 specimens.

Next, we eliminated the possibility of bacterial co-infections at the time of admission based on clinical evaluation, CRP, and white blood cell count, and the available microbiological information. However, empiric antibiotic therapy was started in 33 cases during the stay in the hospital, and bacterial superinfection could not be proven or ruled out by subsequent sampling of these children.

In conclusion, diagnostic advances, including nucleic acid amplification platforms, have greatly improved the detection of respiratory viral pathogens, but the high sensitivity of such molecular methods has raised question about meaning of co-detection of multiple pathogens. One of the major challenges nowadays is distinguishing true infection from asymptomatic carriage [35,36]. In addition, it is difficult to assess the direct impact of HBoV on respiratory tract infections due to its frequent co-detection with other respiratory viruses. A comparison of MT-PCR with quantitative PCR showed that MT-PCR could be powerful tool for distinguishing the true nature of HBoV detection on the basis of amount of virus compared to co-detected pathogens.

## Figures and Tables

**Table 1:** Samples in which only HBoV was detected on MT-PCR with the results of two other tests for those samples.

Multiplex PCR	HBoV Quantitative PCR (Number of Copies/mL)	MT-PCR	MT-PCR HBoV Intensity
HBoV, RSV B	9.70 × 10^6^	HBoV	5
HBoV, PiV 4, AdV	8.40 × 10^5^	HBoV	5
HBoV, PiV 4	2.03 × 10^2^	HBoV	1
HBoV, HRV	5.86 × 10^2^	HBoV	2

**Table 2:** Comparison of results between three methods for samples negative for HBoV on MT-PCR (*N* = 10).

Multiplex PCR	HBoV Quantitative PCR (Number of Copies/mL)	MT-PCR	MT-PCR Sample Adequacy
HBoV, HRV	6.29 × 10^3^	RV/EV *	3
HBoV, HRV, RSV A	37.1	RSV	4
HBoV, HRV, HEV, HCoV	9.71	HCoV, RV/EV, EV *	4
HBoV, HRV	0	RV/EV *	3
HBoV, HEV, AdV	0	RV/EV, EV *	5
HBoV, HRV	0	RV/EV *	3
HBoV, HRV, AdV, PiV 1, PiV 4	0	PiV 4	4
HBoV, RSV A, HCoV	0	RSV, RV/EV *	4
HBoV, HRV, AdV	0	AdV	4
HBoV, AdV	0	0	3

* Note that MT-PCR has an assay for human rhinovirus (RV/EV), which is designed to detect human rhinoviruses groups A to C and human enterovirus groups A to D, and an assay for human enterovirus (EV), which is designed to detect enterovirus groups A to D. If both RV/EV and EV assays are positive, the sample is enterovirus. If only RV/EV assay is positive, the sample is rhinovirus.

**Table 3:** Patients divided in four groups according to amount of HBoV and other respiratory viruses, and localization of infection in the respiratory tract (UTRI—upper respiratory tract infection, LRTI—lower respiratory tract infection).

Group	Quantitative PCR Concentration of HBoV	MT-PCR Intensity of Other Viruses	URTI (*N* of Patients)	LRTI (*N* of Patients)
A	high (more than 10^4^ copies/mL)	4–5 stars	6	8
B	high (more than 10^4^ copies/mL)	1–3 stars	8	6
C	low or negative (less than 10^4^ copies/mL)	4–5 stars	5	9
D	low or negative (less than 10^4^ copies/mL)	1–3 stars	4	9
		Total	23	32

## Data Availability

The data presented in this study are available on request from the corresponding author. The data are not publicly available due to privacy consideration.
